# Supplementary material for: Continued Dispensing: what medications do patients believe should be available?
Source: PeerJ. 2015 May 5;3:e924. doi: 10.7717/peerj.924 (PMC4435445; doi:10.7717/peerj.924)
Supplement: Appendix S1 — Note: This is a shortened version of the questionnaire; it contains only the relevant questions to this manuscript. [file peerj-03-924-s001.docx]

**Appendix 1: Abridged questionnaire for statin and oral contraceptive users views about continued dispensing**

Note: This is a shortened version of the questionnaire; it contains only the relevant questions to this manuscript.

Q1: Which age group do you fall into? *(Please tick the appropriate box)*

| 18-20 | 21-30 | 31-40 | 41-50 | 51-60 | 61-70 | 71-80 | > 80 | Prefer not to disclose |
| --- | --- | --- | --- | --- | --- | --- | --- | --- |
|  |  |  |  |  |  |  |  |  |

Q2: Gender:

| Female | Male | Prefer not to disclose |
| --- | --- | --- |
|  |  |  |

Q3 In which state/territory do you live?

| Australian Capital Territories (ACT) | Queensland (QLD) | New South Wales (NSW) | Northern Territories (NT) | South Australia (SA) | Tasmania (TAS) | Victoria (VIC) | Western Australian (WA) | Prefer not to disclose |
| --- | --- | --- | --- | --- | --- | --- | --- | --- |
|  |  |  |  |  |  |  |  |  |

Q4: What best describes where you live?

| Capital city | Major regional centre | Country town | Rural | Remote | Others; please specify | Prefer not to disclose |
| --- | --- | --- | --- | --- | --- | --- |
|  |  |  |  |  |  |  |

Q11& Q12: Do you suffer from any other chronic disease for which you have used a medicine for more than 6 months? *If YES please tick the answer from the following list:*

|  | Diabetes mellitus |  | High Blood pressure |  | Blood clotting problem |  | Anxiety |  | Depression |
| --- | --- | --- | --- | --- | --- | --- | --- | --- | --- |
|  | Thyroid disorders |  | Chronic pain |  | Asthma |  | Emphysema |  | Chronic bronchitis |
|  | Arthritis |  | Skin chronic problems |  | Reflux & ingestion |  | Glaucoma |  | Other |

Q19: How many times in every 12 months would you prefer to be able to get additional supply of your regular medicine from your pharmacist if you are not able to obtain a new prescription from your doctor before you ran out?

| Once | Twice | Three | Any time my repeats run out and I am not able to get an appointment with my doctor |
| --- | --- | --- | --- |
|  |  |  |  |

Q 21: Pharmacists should be able to provide an additional supply for which of the following prescription medications in the case where it is not convenient to obtain a new prescription from your doctor when all your repeats have run out? *(Please indicate your level of agreement or disagreement)*

| Therapeutic area  (medicines which are used for the following diseases) | Level of Agreement | | | | | |
| --- | --- | --- | --- | --- | --- | --- |
|  | Strongly Disagree | Disagree | Neutral | Agree | Strongly Agree | Prefer not to disclose |
| Oral diabetes medicine |  |  |  |  |  |  |
| Medicines to treat high blood pressure |  |  |  |  |  |  |
| Medicines to treat blood clotting |  |  |  |  |  |  |
| Medicines to treat anxiety |  |  |  |  |  |  |
| Medicines to treat depression |  |  |  |  |  |  |
| Medicines to treat thyroid disorders |  |  |  |  |  |  |
| Medicines to treat chronic pain |  |  |  |  |  |  |
| Medicines to treat asthma |  |  |  |  |  |  |
| Medicines to treat emphysema |  |  |  |  |  |  |
| Medicines to treat chronic bronchitis |  |  |  |  |  |  |
| Medicines to treat arthritis |  |  |  |  |  |  |
| Medicines to treat chronic skin problems |  |  |  |  |  |  |
| Medicines to treat indigestion |  |  |  |  |  |  |
| Medicines to treat glaucoma |  |  |  |  |  |  |
| Others please specify |  | | | | | |
| 1. |  |  |  |  |  |  |
| 2. |  |  |  |  |  |  |
| 3. |  |  |  |  |  |  |
